# AI prediction of extubation success within a novel three-stage liberation framework: development, validation, and implementation of the Stage-3 model

**DOI:** 10.3389/fmed.2025.1725864

**Published:** 2026-01-10

**Authors:** Chin-Ming Chen, Yi-Chen Shao, Chung-Feng Liu, Mei-I Sung, Yu-Ting Shen, Shian-Chin Ko, Chih-Cheng Lai

**Affiliations:** 1 Department of Intensive Care Medicine, Chi Mei Medical Center, Liouying, Taiwan; 2 Department of Medical Research, Chi Mei Medical Center, Tainan, Taiwan; 3 Division of Pulmonary Medicine, Chi Mei Medical Center, Tainan, Taiwan

**Keywords:** artificial intelligence, extubation, intensive care unit, machine learning, mechanical ventilation, predictive model

## Abstract

**Introduction:**

We propose a three-stage liberation decision framework (Stage-1 readiness, Stage-2 SBT success, Stage-3 extubation). While prior tools emphasize earlier stages, Stage-3—deciding whether to remove the tube after SBT—remains under-modeled. This study develops an AI model to predict successful extubation (no reintubation or non-invasive ventilation within 48 h) using routinely collected electronic medical record data, eliminating the need for additional manual bedside measurements.

**Methods:**

Single-center retrospective analysis including 5,202 adults who underwent elective extubation after SBT success. Seven algorithms (Random Forest, LightGBM, XGBoost, Logistic Regression, multilayer perceptron, Voting, Stacking) were trained and evaluated by accuracy, sensitivity, specificity, PPV, NPV, and AUC; interpretability used SHAP; traditional indices (RSBI, etc.) served as comparators. We also implemented a working web-based prototype that verifies the model’s usability and real-world feasibility, providing a foundation for future prospective clinical evaluation.

**Results:**

LightGBM performed best (accuracy 0.797, sensitivity 0.800, specificity 0.763, PPV 0.977, NPV 0.231, AUC 0.861). XGBoost and Voting showed AUC 0.850 with slightly lower accuracies (0.783, 0.771); Stacking AUC 0.829; Random Forest AUC 0.818; MLP and Logistic Regression AUC 0.785 each. SHAP analysis identified SpO₂/FiO₂, department, bilateral lower-limb muscle strength, and dynamic compliance (Cdyn) as most influential predictors of extubation success.

**Discussion:**

Within a three-stage liberation framework, a Stage-3 extubation-focused AI model—particularly LightGBM—outperformed traditional indices and offers explainable, EMR-based predictors to support timely tube removal. A web-based prototype has been developed for future prospective validation.

## 1 Introduction

Invasive mechanical ventilation (IMV) is a crucial life-saving intervention frequently employed in intensive care units (ICU) for patients with respiratory failure. While this intervention is essential for maintaining adequate gas exchange and reducing work of breathing in critically ill patients, the timing of liberation from mechanical ventilation, known as extubation, remains a significant clinical challenge (1). Both premature and delayed extubation can lead to adverse outcomes: premature extubation may result in respiratory failure and the need for reintubation, while prolonged mechanical ventilation is associated with increased risk of ventilator-associated complications, including pneumonia, airway trauma, and muscle weakness (2, 3).

Extubation failure is defined as the need for reintubation within a specified timeframe after elective extubation. While definitions vary across studies (24–72 h), the 48-h window has been widely adopted as a clinically meaningful threshold that captures the majority of extubation-related complications while minimizing confounding from unrelated clinical deterioration (2–5). Studies using this 48-h definition have shown that failed extubation attempts occur in approximately 10–20% of cases and are associated with increased mortality rates, longer ICU stays, and higher healthcare costs (4, 6, 7). Conversely, delayed extubation extends the duration of mechanical ventilation, potentially leading to complications such as ventilator-associated pneumonia, which affects up to 28% of mechanically ventilated patients and carries a mortality rate of 20–50% (8).

As the pathophysiology of extubation failure is not yet fully understood, traditional weaning parameters and clinical judgment alone have shown limited predictive accuracy (5). Given these challenges, there is a pressing need for accurate predictive tools to guide clinicians in determining optimal extubation timing (9, 10). Recent advances in artificial intelligence (AI) and machine learning technologies offer promising opportunities to develop more sophisticated and accurate predictive models for critically ill patients in intensive care units (ICU) (11–16). In prior work, we developed a two-stage AI model addressing earlier weaning decisions (17). Stage-1 (“try-weaning”) predicted the optimal timing for initiating a weaning attempt—transitioning from controlled ventilation to support ventilation modes. Stage-2 (“weaning MV”) predicted the likelihood of successful liberation from mechanical ventilation to spontaneous breathing with supplemental oxygen therapy. Both stages demonstrated high predictive performance (AUC 0.843–0.953 for Stage-1; 0.889–0.944 for Stage-2) and, when implemented as a digital dashboard, reduced intubation time by approximately 21 h (17). However, that approach did not address the final and decisive step: whether a patient who has passed a spontaneous breathing trial (SBT) will succeed after extubation—our Stage-3 focus.

To align prediction with real-world ICU decision points, we extend this concept to a three-stage liberation framework—Stage-1 readiness to wean, Stage-2 SBT success, and Stage-3 extubation. In this study, we focus exclusively on Stage-3, the final and most decisive step of ventilator liberation. Unlike earlier stages that primarily reflect ventilatory mechanics and oxygenation stability, the extubation stage incorporates multidimensional factors such as SpO₂/FiO₂, dynamic compliance (Cdyn), and bilateral lower-limb muscle strength grade (BLL-MSG), which play decisive roles in post-extubation outcomes.

By embedding this third stage into the predictive framework, our study aims to provide a more comprehensive and clinically aligned decision-support approach that mirrors ICU practice and enhances the safety of liberation from mechanical ventilation.

The primary aim of this study was to develop and validate an AI-based Stage-3 model capable of accurately predicting extubation success within 48 h, using variables routinely documented in electronic medical records without requiring additional manual measurements. In addition, we established a real-time prediction prototype system with the built model within the ICU electronic environment to facilitate future prospective evaluation of its clinical feasibility and usefulness in supporting extubation decision-making.

## 2 Methods

### 2.1 Study design, setting, and samples

**Figure 1:**
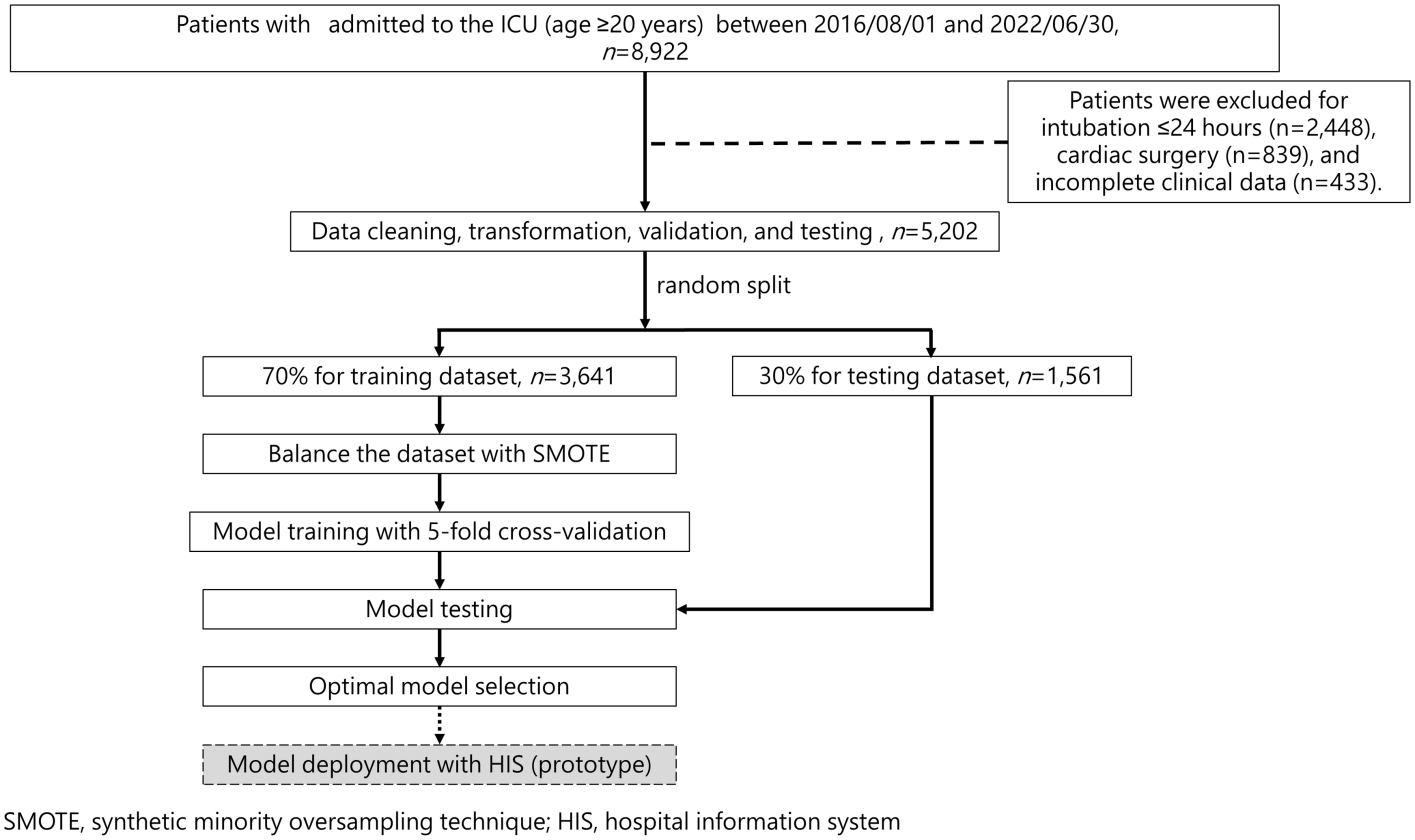
The study flow chart. Dashed borders and gray shading indicate that a prototype application using the selected model was developed and is available for future prospective evaluation.

This retrospective observational study was conducted at Chi Mei Hospital, a large medical center with 1,296 beds, including 95 intensive care unit (ICU) beds. The study subjects were adult patients (aged≥20 years) admitted to the ICU between August 2016 and June 2022 who underwent elective extubation. Elective extubation was defined as clinician-initiated removal of the endotracheal tube following successful completion of a spontaneous breathing trial, as opposed to unplanned extubation (accidental self-extubation or emergent removal). All enrolled patients were endotracheally intubated and receiving invasive positive pressure ventilation. Patients were excluded if they had an intubation duration of ≤24 h, underwent cardiac surgery, had unplanned extubation, or had incomplete clinical data. After screening, the electronic medical records of 5,202 patients who met the elective extubation criteria were included for analysis. The study flow chart is shown in Figure 1. This study was reviewed and approved by the Human Institutional Research Ethics Committee of Chi Mei Hospital (IRB No. 11302–008) and was conducted in full compliance with relevant regulations and ethical guidelines. Due to the retrospective nature of this study, the requirement for patient informed consent was waived.

### 2.2 Feature and outcome variables

Time-zero (T0). We defined T0 *a priori* as the last pre-planned-extubation assessment after SBT success; only predictors available at T0 were used to prevent information leakage.

Predictors. Guided by literature and multidisciplinary consensus, we prespecified 12 candidate predictors available based on two criteria: (1) routine availability in electronic medical records, and (2) no requirement for additional manual measurements or patient cooperation. This design enables automated, real-time bedside prediction. The predictors included: Age, Gender, Department of Admission, presence of Chronic Obstructive Pulmonary Disease (COPD), Oxygen Saturation to Fraction of Inspired Oxygen Ratio (SpO_2_/FiO_2_ ratio), Respiratory Rate–Oxygenation (ROX) index, Dynamic Compliance (Cdyn), Rapid Shallow Breathing index (RSBI), Positive End-Expiratory Pressure (PEEP), Expiratory Tidal Volume (Vte), Peak Inspiratory Pressure (Ppeak), and Bilateral lower limb muscle strength grade (BLL-MSG). All measurements were the most recent values recorded prior to extubation.

Note: Maximum inspiratory pressure (MIP) and maximum expiratory pressure (MEP) were not included as model predictors because they require volitional patient effort and are not routinely documented in real-time electronic records. These parameters were evaluated separately as benchmark comparators (see section 2.5). Additional variables (GCS subscales, suctioning frequency) were evaluated but excluded due to lack of discriminatory value (see Table 1).

**Table 1:** Comparison of demographic and clinical characteristics between successful and failed extubation groups.

Variables	Successful extubation		*P*-value
	No	Yes	
	*N* = 381	*N* = 4,821	
Age, mean (SD)	68.50 (14.14)	63.98 (15.65)	<0.001
Gender, n (%)			
Female	173 (45.41)	1,606 (33.31)	<0.001
Male	208 (54.59)	3,215 (66.69)	
Department, n (%)			
Internal medicine	242 (63.52)	2,286 (47.42)	<0.001
Surgical department	139 (36.48)	2,535 (52.58)	
History_COPD, n (%)	65 (17.06)	471 (9.77)	<0.001
BLL-MSG ≥ 8, n (%)	172 (45.14)	2,448 (50.78)	0.039
SpO_2_/FiO_2_ ratio, mean (SD)	367.34 (37.31)	394.42 (5.02)	<0.001
ROX index, mean (SD)	22.18 (6.71)	25.05 (7.27)	<0.001
PEEP, mean (SD)	5.58 (1.07)	5.26 (0.78)	<0.001
Vte, mean (SD)	445.08 (110.31)	491.76 (117.00)	<0.001
Ppeak, mean (SD)	16.09 (2.12)	15.35 (1.83)	<0.001
Cdyn, mean (SD)	42.38 (11.19)	49.41 (13.45)	<0.001
RSBI, mean (SD)	40.63 (16.09)	33.53 (14.15)	<0.001
GCS_eyes open, mean (SD)	3.57 (0.61)	3.53 (0.63)	0.221
GCS_motor response, mean (SD)	5.80 (0.55)	5.76 (0.58)	0.260
Suction frequency, mean (SD)	5.71 (4.27)	5.38 (4.01)	0.145

FiO_2_, Inspired oxygen fraction; PEEP, Positive end-expiratory pressure; Vte, Expiratory tidal volume; Ppeak, Peak inspiratory pressure; RSBI, Rapid shallow breath index; ROX, Respiratory rate Oxygenation index; Cdyn, Dynamic Compliance; BLL-MSG, Bilateral lower limb muscle strength grade; GCS, Glasgow Coma Scale. Data are presented as mean (SD) for continuous variables and n (%) for categorical variables.

The primary outcome was extubation success, defined as no reintubation and no non-invasive ventilatory support within 48 h following elective endotracheal tube removal. Patients requiring reintubation or any non-invasive ventilatory support within 48 h after elective extubation were classified as extubation failures.

### 2.3 Model building and performance evaluation

Machine learning modeling was performed using Python on the Anaconda platform. Traditional models were implemented using Scikit-learn. Raw ICU electronic medical record data, after cleaning and pre-processing, was randomly divided into a 70% training set and a 30% test set. To address data imbalance due to a low proportion of minority cases, the training set was oversampled using the SMOTE technique. During the modeling phase, various algorithms were employed, including Logistic Regression (LR), Random Forest (RF), LightGBM, XGBoost, MLPClassifier, Voting and Stacking Feature importance analysis was performed using SHAP to enhance model interpretability. Grid Search and 5-fold cross-validation (CV) were used to adjust hyperparameters in the training data set, while the test set was used for final performance verification. Model evaluation metrics include accuracy, sensitivity, specificity, positive predictive value (PPV), negative predictive value (NPV), and area under the receiver operating characteristic curve (AUC), with AUC serving as the primary basis for optimal model selection. Given the clinical importance of identifying patients at high risk of extubation failure, NPV—which reflects the model’s ability to correctly identify high-risk patients when predicting failure—was recognized as a critical performance metric despite the anticipated challenge posed by class imbalance (low proportion of extubation failures in the dataset).

### 2.4 Statistical analysis and software version

Before model development, descriptive statistics were used to summarize patient characteristics. Continuous variables were first examined for normality and were reported as the mean and standard deviation (SD), which was deemed appropriate for the observed data distributions. Categorical variables were presented as frequency counts with percentages. Baseline characteristic differences between the successful and failure groups were evaluated using Student’s *t*-test for continuous variables and Pearson’s chi-squared test or Fisher’s exact test for categorical variables. A *p*-value <0.05 was considered to show statistical significance. All machine learning modeling was performed using the Python (version 3.7.6) programming language on the Anaconda platform (version 4.8.3), primarily within a Jupyter Notebook environment. Traditional machine learning models were implemented using the Scikit-learn library (version 0.22.2.post1), and gradient boosting methods employed LightGBM (version 2.3.1) and XGBoost (version1.1.1).

### 2.5 Comparison with traditional weaning parameters

To benchmark the performance of our Stage-3 AI model, we compared it against traditional weaning parameters commonly used in clinical practice for extubation decision-making. These conventional parameters were obtained through standardized manual bedside measurements at the same time point as the AI model predictors.

RSBI was manually measured using a handheld spirometer (Haloscale Wright Respirometer) over 1 min to derive respiratory rate to tidal volume ratio (breaths/min/L). This manual measurement approach was employed to reflect real-world clinical practice, as opposed to relying exclusively on automated ventilator-derived data. Maximum Inspiratory Pressure (MIP) and Maximum Expiratory Pressure (MEP) were manually measured using a handheld pressure gage to assess respiratory muscle strength. MIP reflects the patient’s maximum capacity to generate negative inspiratory pressure, while MEP indicates maximum expiratory muscle force. These parameters were evaluated both individually (RSBI alone; MIP and MEP in combination) and collectively (RSBI, MIP, and MEP combined). Rather than applying predetermined clinical cutoff thresholds, continuous measurements were utilized as predictors in logistic regression models. These models were trained on the same training dataset (70% of the total cohort) and evaluated on the same test dataset (30% of the cohort, *n* = 1,561) as the machine learning models. This methodological approach enables data-driven optimal threshold determination and ensures standardized comparison with our AI models. Performance metrics, including accuracy, sensitivity, specificity, positive predictive value (PPV), negative predictive value (NPV), and area under the receiver operating characteristic curve (AUC), were calculated for each traditional parameter approach and compared with the AI model predictions.

### 2.6 Model selection and implementation of a web-based predictive prototype

The model with the highest AUC value, LightGBM model, was selected as the optimal Stage-3 model. We implemented it as a web-based, point-of-care prototype application on the hospital intranet, designed to support physicians and respiratory therapists at T0 and serve as a foundation for future prospective clinical evaluation.

## 3 Results

### 3.1 Baseline characteristics of included patients

A total of 5,202 patients were included. The mean age was 64.31 ± 15.59 years, and the cohort was predominantly male (65.8%). Nearly half of the patients were admitted through internal medicine (48.6%), and 51.4% through surgical departments. COPD was present in 10.3% of patients. and 50.4% had BLL-MSG ≥ 8 (Table 2). Ventilator parameters indicated low support levels: mean PEEP 5.29 ± 0.81 cmH₂O, tidal volume 488.34 ± 117.15 mL, Ppeak 15.41 ± 1.87 cmH₂O, and Cdyn 48.89 ± 13.42 mL/cmH₂O. Mean SpO₂/FiO₂ ratio was 392.44 ± 13.23, ROX index 24.84 ± 7.27, and RSBI 34.05 ± 14.42 breaths/min/L. Baseline comparisons between successful and failed extubation groups are presented in Table 1.

**Table 2:** The demographic and clinical characteristics of patients.

Variables	Total patients
	*n* = 5,202
Age, mean (SD)	64.31 (15.59)
Gender, n (%)	
Female	1779 (34.20)
Male	3,423 (65.80)
Department, n (%)	
Internal medicine	2,528 (48.60)
Surgical department	2,674 (51.40)
COPD, n (%)	536 (10.30)
BLL-MSG ≥ 8, n (%)	2,620 (50.37)
SpO_2_/FiO_2_ ratio, mean (SD)	392.44 (13.23)
ROX index, mean (SD)	24.84 (7.27)
PEEP, mean (SD)	5.29 (0.81)
Vte, mean (SD)	488.34 (117.15)
Ppeak, mean (SD)	15.41 (1.87)
Cdyn, mean (SD)	48.89 (13.42)
RSBI, mean (SD)	34.05 (14.42)

FiO_2_, Inspired oxygen fraction; PEEP, Positive end-expiratory pressure; Vte, Expiratory tidal volume; Ppeak, Peak inspiratory pressure; RSBI, Rapid shallow breath index; ROX, Respiratory rate Oxygenation index; Cdyn, Dynamic Compliance; BLL-MSG, Bilateral lower limb muscle strength grade. Data are presented as mean (SD) for continuous variables and n (%) for categorical variables. *p*-values derived from Student’s *t*-test (continuous variables) or chi-square/Fisher’s exact test (categorical variables).

### 3.2 Predictive performance of each model

**Figure 2:**
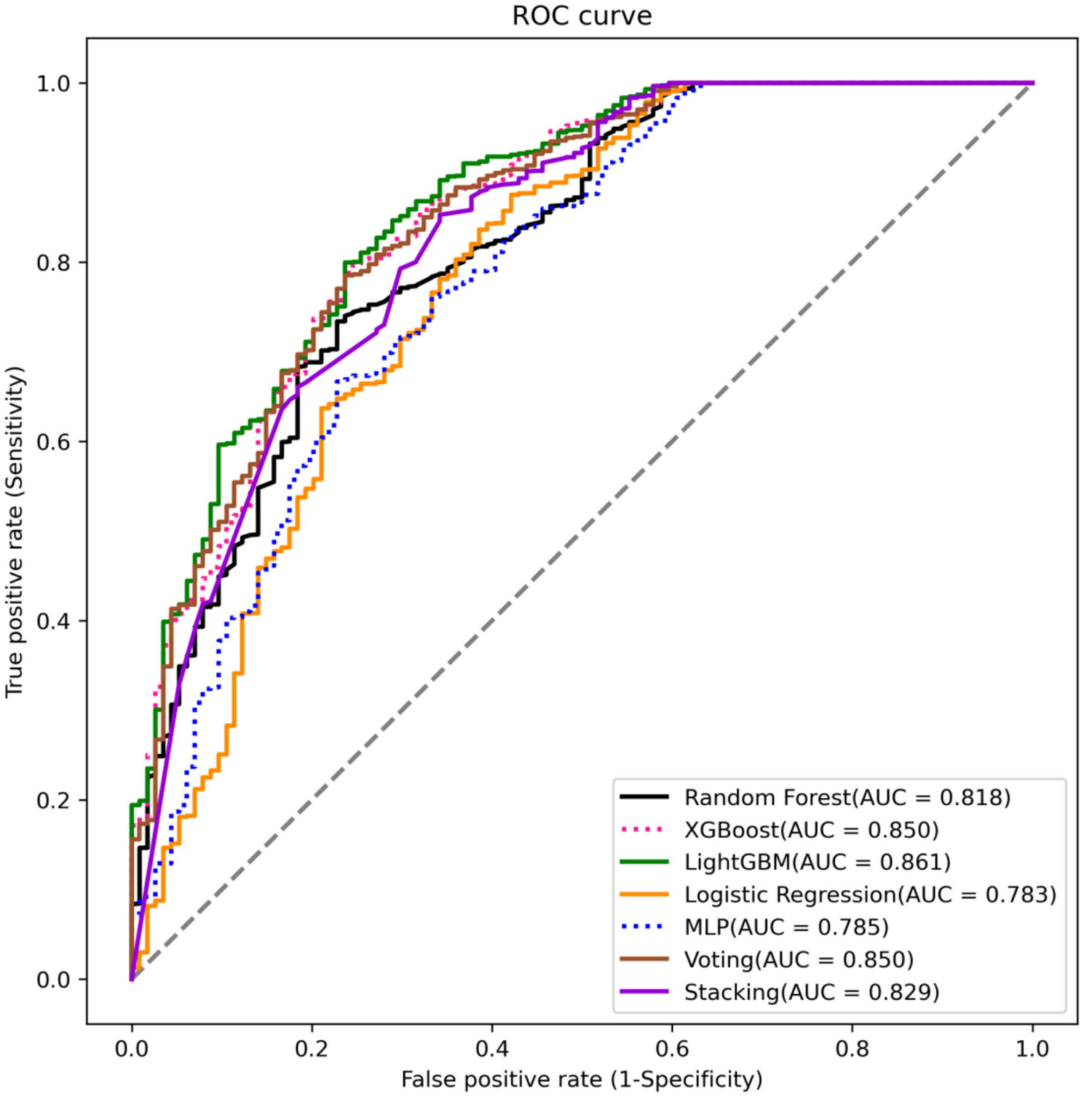
Receiver operating characteristic (ROC) curves for extubation success prediction models.

Among the seven machine learning algorithms evaluated, LightGBM demonstrated the best overall performance with the highest accuracy (0.797), sensitivity (0.800), and area under the curve (AUC) of 0.861 (Table 3 and Figure 2). This was followed by XGBoost and Voting, which showed comparable performance metrics (AUC of 0.850 for both). The Stacking logistic regression model achieved moderate performance with an AUC of 0.829 and an accuracy of 0.725, while the Logistic Regression showed the lowest performance across AUC: 0.756. Notably, all models maintained relatively high positive predictive values (PPV) ranging from 0.968 to 0.977, indicating strong reliability when predicting extubation success. However, negative predictive values (NPV 0.155–0.231) were substantially lower across all algorithms, indicating limited ability to reliably identify patients at high risk of extubation failure when the model predicts failure. This low NPV reflects the inherent challenge of predicting rare events in imbalanced datasets, where extubation failures (*n* = 381, 7.3%) were substantially outnumbered by successes (*n* = 4,821, 92.7%). The specificity values were consistent across most models (approximately 0.72), except for Logistic Regression which showed slightly lower specificity (0.702) (Table 3).

**Table 3:** Predictive performance of each model.

Algorithm	Accuracy	Sensitivity	Specificity	AUC	PPV	NPV
Logistic regression	0.714	0.715	0.702	0.783	0.968	0.162
Random forest	0.746	0.746	0.746	0.818	0.974	0.188
LightGBM	0.797	0.800	0.763	0.861	0.977	0.231
MLPClassifier	0.693	0.690	0.719	0.785	0.969	0.155
XGBoost	0.783	0.785	0.763	0.850	0.977	0.219
Voting	0.771	0.772	0.763	0.850	0.976	0.209
Stacking	0.725	0.725	0.728	0.829	0.971	0.173

MLP, multilayer perceptron.

### 3.3 SHapley additive exPlanations analysis

**Figure 3:**
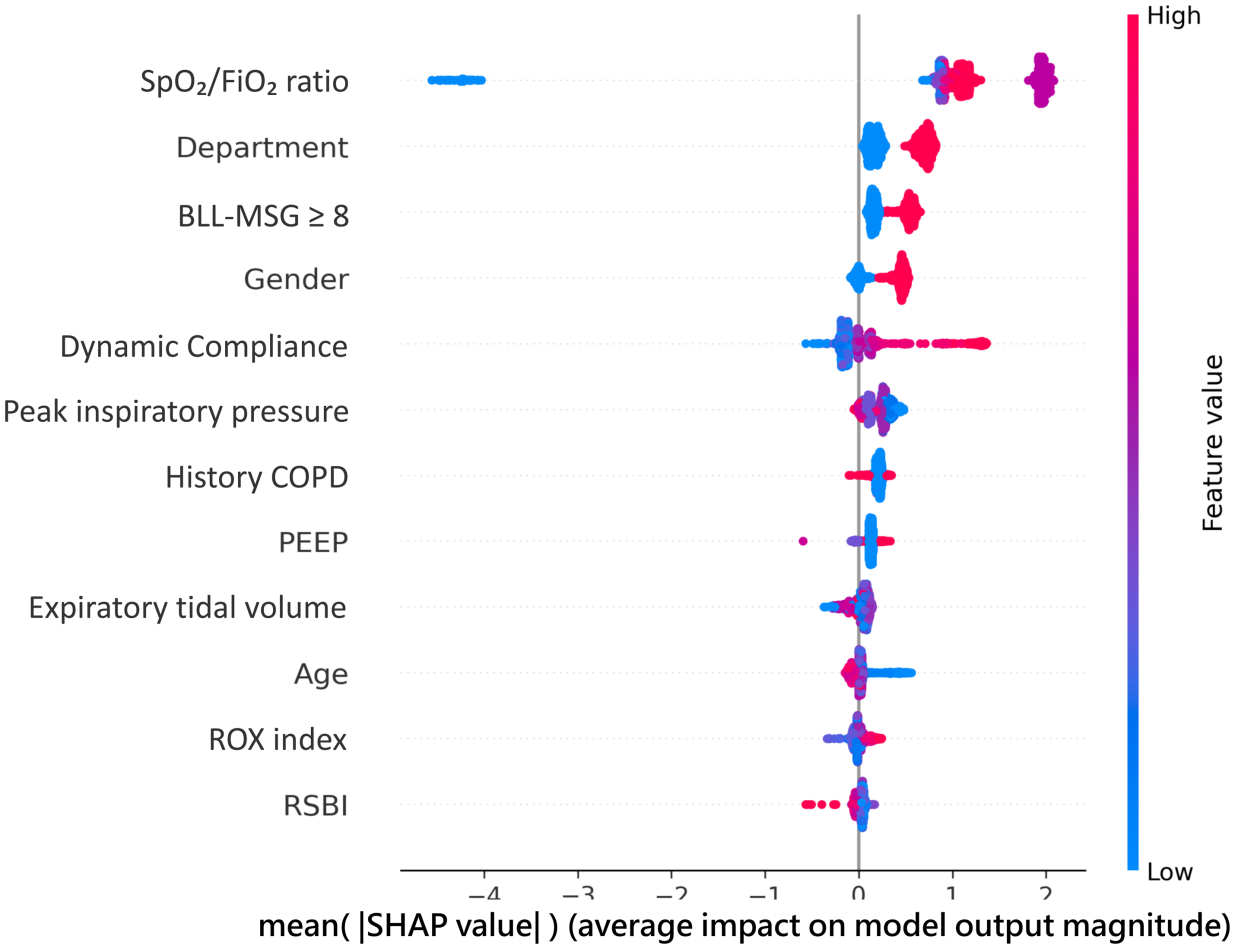
SHAP summary plot for the LightGBM model. Features are ranked by mean absolute SHAP value. Each point represents one patient; horizontal position indicates contribution to prediction (positive = toward success); color indicates feature value (red = high, blue = low).

**Figure 4:**
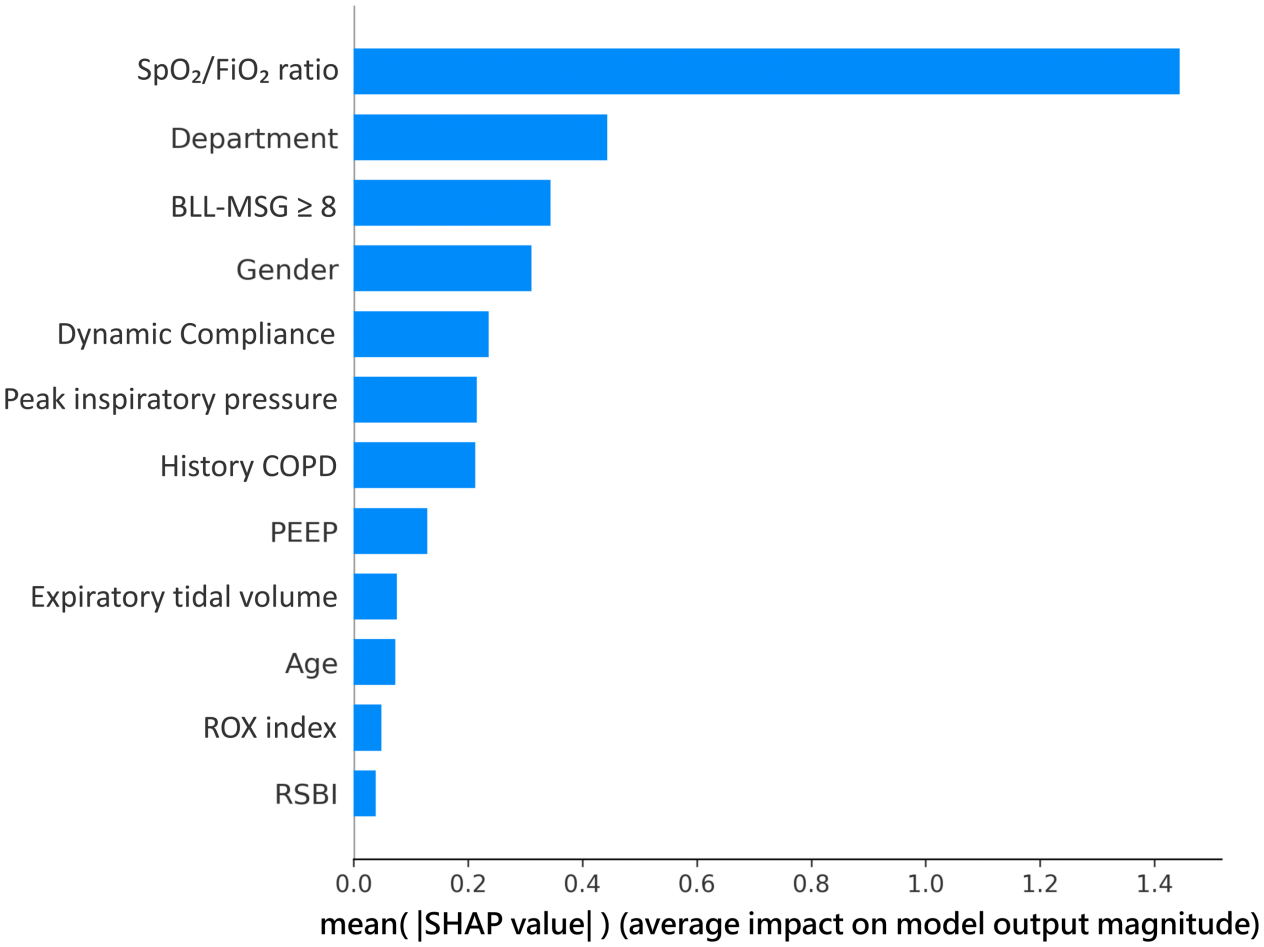
SHAP feature importance ranking for the LightGBM model. Bar length represents mean absolute SHAP value across all patients.

The SHAP (SHapley Additive exPlanations) analysis illustrated the relative importance of individual features in the LightGBM model’s predictions for extubation outcomes (Figure 3: global feature importance/beeswarm; Figure 4: representative dependence plots with SHAP values, including overlaid marginal trends). The SpO₂/FiO₂ ratio emerged as the most influential predictor, with higher values associated with an increased probability of successful extubation. The department of admission also contributed significantly, as patients admitted under surgical services showed a higher likelihood of successful extubation compared with those under internal medicine. Bilateral lower-limb muscle strength (BLL-MSG), gender, and Cdyn followed as important predictors, while Ppeak, COPD, and PEEP demonstrated moderate influence. In contrast, features like the ROX index and RSBI had a smaller impact on the model output, reflected in their lower SHAP values.

### 3.4 Comparison between traditional parameter and AI model

Across evaluation metrics, the AI model (Stage-3 LightGBM at the prespecified clinical operating threshold) outperformed traditional parameters (Table 4). The AI model achieved the highest accuracy (0.797), sensitivity (0.800), specificity (0.763), and AUC (0.861), significantly outperforming traditional parameters. Among the conventional methods, the combination of RSBI, MEP, MIP, and cuff leak test showed moderate performance (accuracy: 0.577, AUC: 0.616), while RSBI alone demonstrated limited predictive capability (accuracy: 0.639, AUC: 0.637) (Table 4). The combination of MEP and MIP exhibited the lowest performance among all methods (accuracy: 0.508, AUC: 0.519). Notably, all methods maintained relatively high PPVs (0.929–0.977) but showed considerably lower NPVs (0.075–0.231).

**Table 4:** Comparison between traditional parameters and AI model for predicting successful extubation rate.

Prediction method	Accuracy	Sensitivity	Specificity	AUC	PPV	NPV
AI model algorithm (LightGBM)	0.797	0.800	0.763	0.861	0.977	0.231
*Manual measurement of RSBI	0.639	0.645	0.561	0.637	0.949	0.111
Manual measurement of MIP and MEP	0.508	0.508	0.509	0.519	0.929	0.075
*Manual measurement of RSBI, MIP, and MEP	0.577	0.578	0.57	0.616	0.945	0.096

*The traditional Rapid Shallow Breathing Index (RSBI) was manually measured using a handheld spirometer (Haloscale Wright Respirometer). MIP, Maximum Inspiratory Pressure; MEP, Maximum Expiratory Pressure; AUC, Area Under the Receiver Operating Characteristic Curve; PPV, Positive Predictive Value; NPV, Negative Predictive Value.

### 3.5 Real-world presentation of the Stage-3 AI predictive prototype system

**Figure 5:**
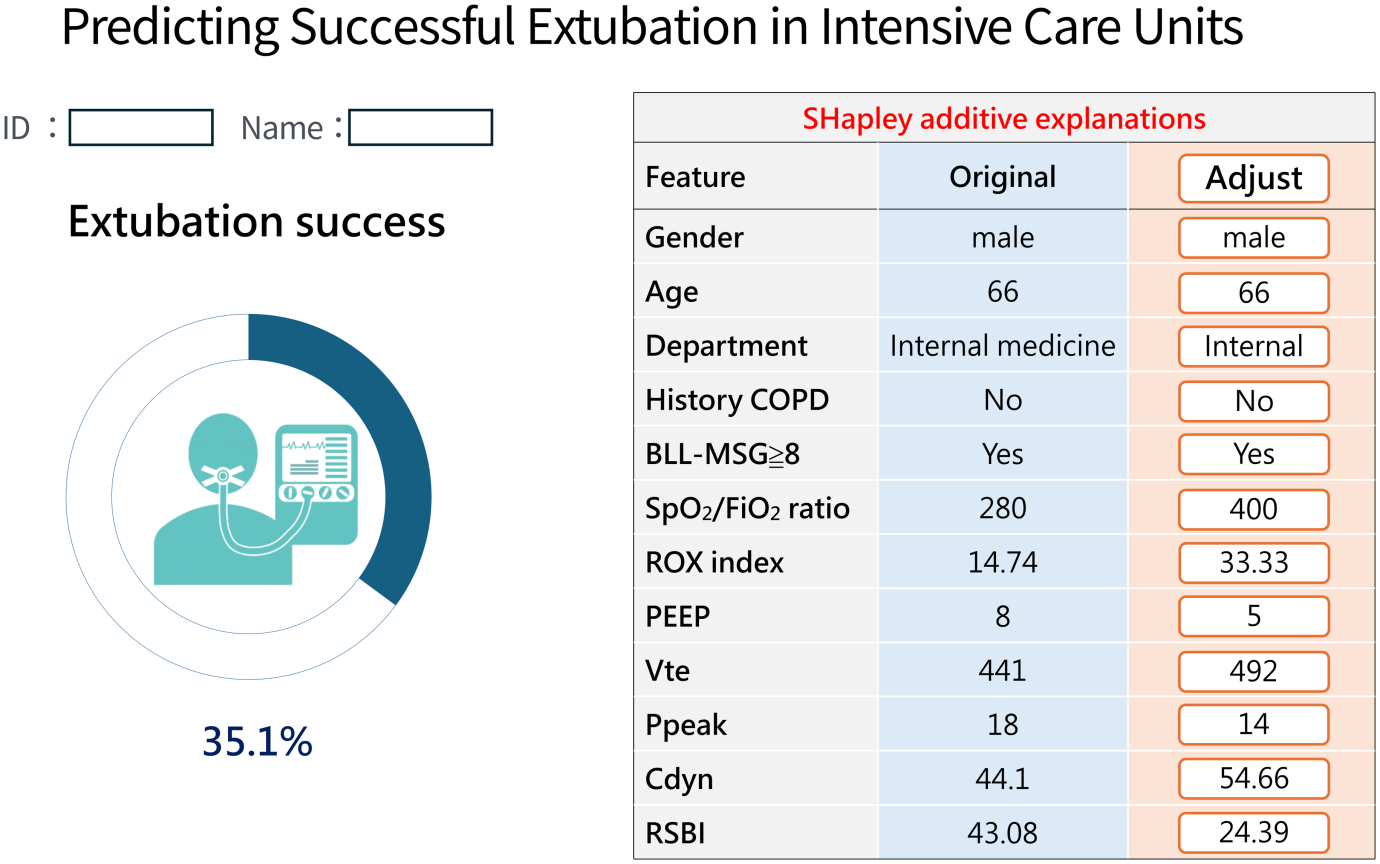
Real-time extubation prediction application interface: Example of a case with low predicted success probability.

**Figure 6:**
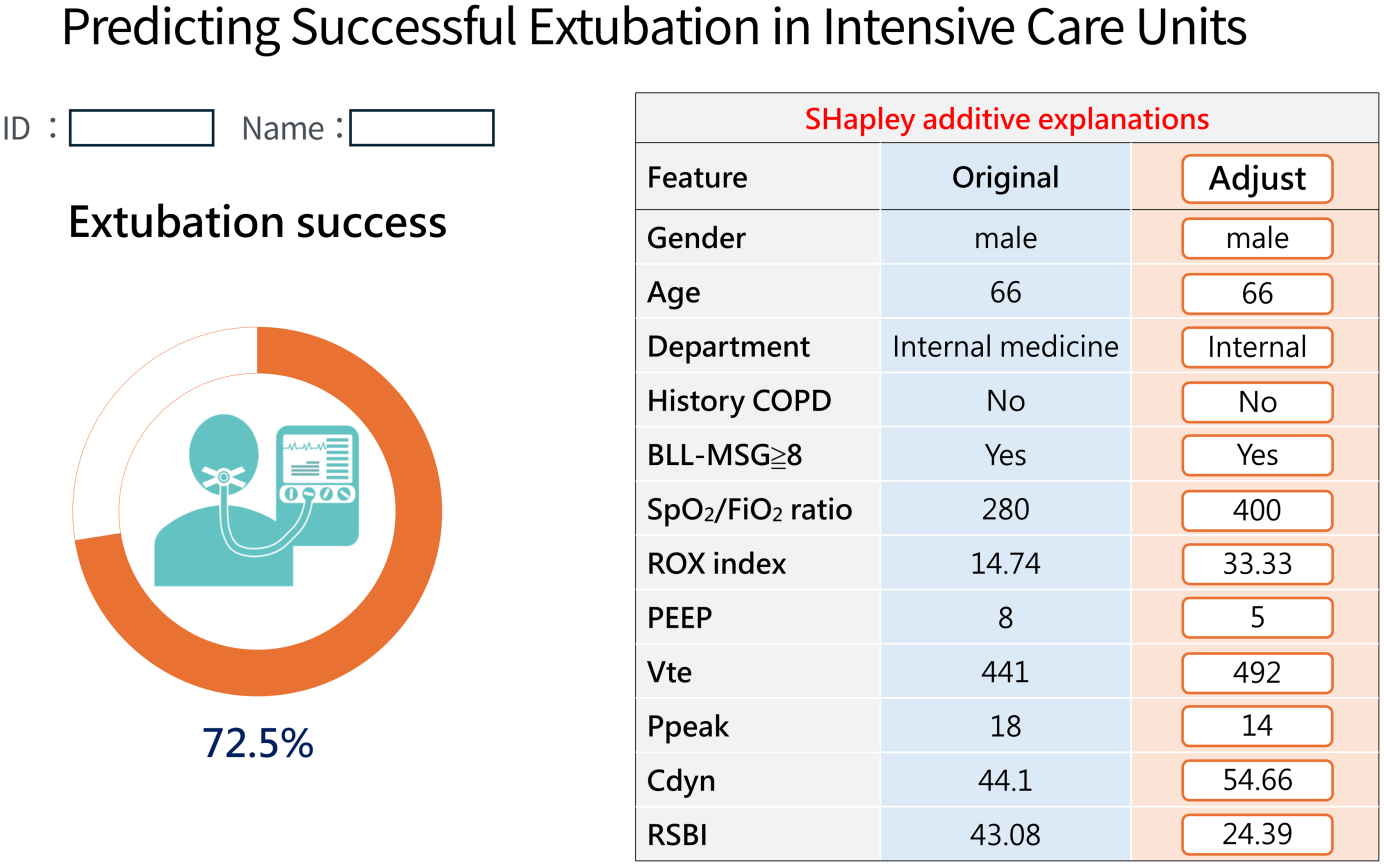
Real-time extubation prediction application interface: Example of a case with high predicted success probability (after manually adjusting some feature values).

To demonstrate the potential clinical application of the Stage-3 model, we illustrate its use with a representative ICU case. As shown in Figure 5, a 66-year-old man without COPD (internal medical service) had BLL-MSG ≥ 8. At T0, physiologic parameters were: SpO₂/FiO₂ 280, ROX 14.74, PEEP 8 cmH₂O, Vte 441 mL, Ppeak 18 cmH₂O, Cdyn 44.1 mL/cmH₂O, and RSBI 43.08, yielding a predicted success probability of 35.1% (<50% indicates low probability). After manually entering improved values—SpO₂/FiO₂ 400, ROX 33.33, PEEP 5 cmH₂O, Vte 492 mL, Ppeak 14 cmH₂O, Cdyn 54.66 mL/cmH₂O, and RSBI 24.39,the predicted probability increased to 72.5%(≥50% indicates high probability of successful extubation) (Figure 6). This demonstrates that improvements in physiological indices are associated with a higher likelihood of successful extubation.

## 4 Discussion

### 4.1 Main findings and contributions

A major innovation of this study lies in explicitly defining extubation as a distinct third stage in the liberation process, rather than subsuming it under the broader concept of weaning. Our previous two-stage framework (17) successfully predicted the transitions from control to support ventilation and from support ventilation to spontaneous breathing. However, it did not address the most clinically decisive step—whether the patient could maintain airway protection and adequate spontaneous breathing after extubation.

To fill this critical gap, we developed a Stage-3, extubation-specific AI model, with LightGBM achieving the best performance (accuracy = 0.797; AUC = 0.861) and demonstrated superior discrimination compared with conventional parameters including RSBI and respiratory muscle strength measures. The high PPV (0.977) supports confident identification of patients likely to succeed, potentially avoiding unnecessary prolongation of mechanical ventilation. However, the low NPV (0.231) indicates limited ability to reliably identify high-risk patients, reflecting class imbalance and feature limitations discussed in section 4.6. The model’s overall performance advantage reflects its capacity to integrate diverse clinical variables and capture non-linear associations relevant to post-extubation outcomes.

### 4.2 Clinical significance of department of admission

SHAP analysis identified admission department as the second most important predictor. Department is not merely a superficial administrative variable, but rather an indicator representing different patient phenotypes with varying pathophysiological characteristics and extubation risk profiles.

As shown in Table 1, the proportion of internal medicine patients in the extubation failure group was significantly higher than that in the surgical group (63.52% vs. 47.42%, *p* < 0.001), and they were older, had a higher prevalence of chronic obstructive pulmonary disease (COPD), and had poorer oxygenation and respiratory reserve.

Literature indicates that the extubation failure rate in internal medicine ICUs is approximately 10–20%, higher than the 5–10% in surgical ICUs (18–20). This difference mainly stems from the patients’ baseline status: internal medicine patients often have chronic cardiopulmonary diseases, longer disease duration, and require longer-term ventilator support; surgical patients mostly suffer from respiratory failure due to acute and reversible causes, and their baseline respiratory function is relatively preserved. The mortality rate of internal medicine patients with extubation failure is also significantly higher (18, 19). Although the admission department is not a direct physiological measurement, it encompasses clinical information such as baseline health status, disease severity, and recovery pattern, and can be regarded as an integrated variable.

### 4.3 Model interpretability: key predictors identified by SHAP analysis

SHAP analysis provided interpretability by highlighting the key factors influencing extubation outcomes. The SpO₂/FiO₂ ratio emerged as the most influential predictor. Unlike PaO₂/FiO₂, which requires arterial blood gas sampling, SpO₂/FiO₂ can be easily obtained at the bedside, making it practical for real-time prediction (21). Bilateral lower-limb muscle strength grade (BLL-MSG) was incorporated as a surrogate indicator for respiratory muscle reserve and cough effectiveness, which are difficult to capture in structured EMR data. Peripheral muscle strength has been associated with global skeletal muscle function and extubation outcomes. Prior studies have shown that limb weakness is an independent predictor of extubation failure (22) and correlates with post-extubation prognosis and functional recovery (23). These results emphasize that both oxygenation efficiency and muscle strength play crucial and complementary roles in extubation outcomes.

### 4.4 Comparison with conventional parameters

Although traditional weaning indices such as RSBI remain widely used in clinical practice (24–26), our findings demonstrated that RSBI alone had limited predictive ability (accuracy = 0.639, AUC = 0.637). Even when combined with other conventional indices, performance did not improve (accuracy = 0.577, AUC = 0.616). This underscores the limitations of relying solely on traditional weaning parameters.

This counterintuitive finding—RSBI alone being superior to combined with MIP and MEP—may reflect the inherent limitations of volitional respiratory muscle strength testing. RSBI assessment involves passive observation during spontaneous breathing, whereas MIP and MEP require maximal volitional efforts against resistance; therefore, they are highly dependent on patient compliance, effort, and the stability of respiratory effort. These effort-dependent measurements introduce significant variability, which may mask the predictive value when used in combination. In contrast, our AI model utilizes variables routinely recorded in electronic medical records (EMRs) to achieve superior performance (accuracy 0.797, AUC 0.861) without requiring additional manual measurements or patient cooperation. All predictive metrics—including SpO₂/FiO₂, dynamic lung compliance, ventilator parameters, and muscle strength assessed in routine clinical examinations—are available without the need for specialized equipment or active patient testing. This EMR-based approach eliminates measurement errors and enables continuous, automated predictions without increasing patient burden or consuming clinician time.

Importantly, this AI model is not intended to replace clinician judgment but rather to interpret conventional metrics within a broader, stage-specific decision-making framework. This is a key advantage of our Stage 3 approach: leveraging existing EMR data to support extubation decisions without disrupting clinical workflows.

### 4.5 Clinical implications

The findings have direct implications for real-world clinical decision-making. The Stage-3 AI model, when prospectively integrated into a real-time prediction system, could enable continuous monitoring of intubated patients, provide instant visualization of extubation readiness, and alert clinicians to optimal weaning timing.

This integration would bridge the gap between predictive analytics and bedside care, transforming AI models from retrospective research tools into prospective, actionable decision support systems. By displaying evidence-based predictions alongside vital signs and ventilator parameters, such a dashboard could enhance situational awareness, support multidisciplinary communication among physicians, respiratory therapists, and nurses, and help reduce clinical uncertainty during daily ICU rounds. In practice, such a system may help avoid unnecessary prolongation of mechanical ventilation, shorten ICU length of stay, and lower the risk of ventilator-associated complications. Furthermore, continuous feedback and utilization data generated through prospective use would provide opportunities for ongoing model refinement and performance monitoring, ensuring that the AI predictive system remains adaptive, transparent, and aligned with patient safety and quality-of-care objectives.

The development and validation of this Stage-3 model represents a foundational step toward data-informed, patient-centered precision care in critical medicine. However, prospective implementation studies are necessary to validate these anticipated benefits and assess real-world clinical impact.

### 4.6 Limitations

This study has several limitations. First, this was a single-center retrospective study; generalizability to other ICUs with different patient characteristics and clinical practices remains uncertain, and clinical impact requires prospective validation.

Second, the low NPV (0.155–0.231) indicates limited ability to identify patients at high risk of extubation failure. This reflects both class imbalance (7.3% failure rate) and feature limitations. Key Stage-3 variables, including direct cough strength assessment and airway protection maneuvers—were not routinely documented in structured EMR data. We evaluated available surrogates: neurologic status (GCS subscales) and suctioning frequency as a proxy for secretion burden; however, neither showed significant differences between groups (Table 1). Notably, threshold adjustment can improve NPV: lowering the threshold from 0.85 to 0.65 increases NPV from 0.231 to 0.725 (sensitivity 0.987), allowing clinicians to customize the operating point based on clinical priorities (Supplementary Table 1). Future development should prioritize prospective collection of standardized airway assessments.

Despite low NPV, the model retains utility as an early alert tool. With high PPV (0.977), it can prompt evaluation of extubation readiness in patients predicted to succeed, reducing unnecessary prolongation of mechanical ventilation.

Third, SMOTE was employed to address class imbalance. Although this improves sensitivity for rare events, synthetic patterns may not fully capture real-world heterogeneity. External validation without oversampling is essential to confirm performance.

### 4.7 Future directions

Future work should focus on enhancing model performance, validating generalizability, and enabling responsible clinical implementation.

First, efforts should aim to improve the model’s negative predictive value by incorporating additional clinical and physiological variables—such as airway protection, secretion burden, cough strength, sedation depth, and delirium status—to better identify patients at risk of extubation failure.

Second, external and multicenter validation across diverse ICUs and patient subgroups is essential to assess the model’s robustness and transferability.

Third, prospective implementation studies should be conducted to evaluate the model’s real-world impact on reintubation rates, ICU length of stay, ventilator-free days, and healthcare costs.

In parallel, a dashboard system should be developed to visualize the model’s outputs and provide real-time predictions for all intubated patients, assisting clinicians in determining the optimal timing for ventilator weaning and extubation.

Finally, model deployment should adhere to Responsible AI principles, incorporating ongoing performance monitoring, transparency reporting, and fairness auditing to ensure regulatory readiness (e.g., TFDA and ISO 13485 compliance) and sustainable clinical adoption.

## 5 Conclusion

Within a three-stage liberation framework, we developed a Stage-3 (extubation)–focused AI model that, using LightGBM, delivered superior discrimination to traditional weaning parameters for predicting 48-h post-extubation success. By integrating readily available bedside variables and providing explainable outputs, the model functions as a decision-support tool that complements—rather than replaces—clinical judgment. Nonetheless, low negative predictive values and the single-center, retrospective design underscore the need for further refinement (e.g., airway-protection and secretion burden measures) and multicenter prospective validation. Future studies should assess clinical impact—including reintubation rates, ICU length of stay, and costs—following real-world implementation and ongoing calibration monitoring.

## Data Availability

The original contributions presented in the study are included in the article/Supplementary material, further inquiries can be directed to the corresponding authors.
