# Supplementary material for: AI prediction of extubation success within a novel three-stage liberation framework: development, validation, and implementation of the Stage-3 model
Source: Front Med (Lausanne). 2026 Jan 10;12:1725864. doi: 10.3389/fmed.2025.1725864 (PMC12870664; doi:10.3389/fmed.2025.1725864)
Supplement: Supplementary file 1 [file Table_1.pdf]

Supplementary Table 1. Performance Metrics of LightGBM Model Across Different Decision Thresholds

Threshold	Accuracy	Sensitivity	Specificity	AUC	PPV	NPV
0.5 [algorithm default]	0.956	0.999	0.395	0.861	0.954	0.999
0.6	0.955	0.997	0.412	0.861	0.956	0.922
0.65	0.947	0.987	0.439	0.861	0.957	0.725
0.7	0.933	0.969	0.482	0.861	0.959	0.550
0.75	0.914	0.947	0.491	0.861	0.959	0.421
0.8	0.876	0.896	0.614	0.861	0.967	0.318
0.85	0.797	0.800	0.763	0.861	0.977	0.231
0.9	0.630	0.611	0.877	0.861	0.984	0.151

*AUC, Area Under the Receiver Operating Characteristic Curve; PPV, Positive Predictive Value; NPV, Negative Predictive Value.
